# Supplementary material for: Resolving the full spectrum of human genome variation using Linked-Reads
Source: Genome Res. 2019 Apr;29(4):635–45. doi: 10.1101/gr.234443.118 (PMC6442396; doi:10.1101/gr.234443.118)
Supplement: Supplemental Material [file supp_gr.234443.118_Supplemental_longranger-2.2.zip › longranger-2.2.tar.bz2!/lr2.2-public/lib/stan/cmdstan/stan/doc/api/html/univariate_2continuous_8hpp.html]

Stan: src/stan/prob/distributions/univariate/continuous.hpp File Reference


|  |  |
| --- | --- |
|  | Stan  2.5.0  probability, sampling & optimization |


- Main Page
- Related Pages
- Namespaces
- Classes
- Files

- File List
- File Members

All Classes Namespaces Files Functions Variables Typedefs Enumerations Enumerator Friends Macros Pages

- src
- stan
- prob
- distributions
- univariate

continuous.hpp File Reference

`#include <stan/prob/distributions/univariate/continuous/beta.hpp>`  
`#include <stan/prob/distributions/univariate/continuous/cauchy.hpp>`  
`#include <stan/prob/distributions/univariate/continuous/chi_square.hpp>`  
`#include <stan/prob/distributions/univariate/continuous/double_exponential.hpp>`  
`#include <stan/prob/distributions/univariate/continuous/exponential.hpp>`  
`#include <stan/prob/distributions/univariate/continuous/exp_mod_normal.hpp>`  
`#include <stan/prob/distributions/univariate/continuous/frechet.hpp>`  
`#include <stan/prob/distributions/univariate/continuous/gamma.hpp>`  
`#include <stan/prob/distributions/univariate/continuous/gumbel.hpp>`  
`#include <stan/prob/distributions/univariate/continuous/inv_chi_square.hpp>`  
`#include <stan/prob/distributions/univariate/continuous/inv_gamma.hpp>`  
`#include <stan/prob/distributions/univariate/continuous/logistic.hpp>`  
`#include <stan/prob/distributions/univariate/continuous/lognormal.hpp>`  
`#include <stan/prob/distributions/univariate/continuous/normal.hpp>`  
`#include <stan/prob/distributions/univariate/continuous/pareto.hpp>`  
`#include <stan/prob/distributions/univariate/continuous/pareto_type_2.hpp>`  
`#include <stan/prob/distributions/univariate/continuous/rayleigh.hpp>`  
`#include <stan/prob/distributions/univariate/continuous/scaled_inv_chi_square.hpp>`  
`#include <stan/prob/distributions/univariate/continuous/skew_normal.hpp>`  
`#include <stan/prob/distributions/univariate/continuous/student_t.hpp>`  
`#include <stan/prob/distributions/univariate/continuous/uniform.hpp>`  
`#include <stan/prob/distributions/univariate/continuous/von_mises.hpp>`  
`#include <stan/prob/distributions/univariate/continuous/weibull.hpp>`

Go to the source code of this file.


---

[ Stan Home Page ]

*© 2011–2014,
Stan Development Team.*
